# Interlobitis in the Tuberculous

**Published:** 1918-09

**Authors:** 


**Interlobitis in the Tuberculous.** Charles Sabourin, Presse Med., Feb. 19, employs this term, indefensible etymologically, to call attention to localized inflammations or the pleura. He considers membranous pleurisy in form of shirt buttons as serious especially in weak individuals. Metapneumonic adhesive pleurisies cause adhesions of the lobes to each other and may surround the pericardium. Serous effusions are almost always absorbed, and may prove benign in facilitating the healing of pre-existing tubercular lesions. Every crisis of bacillar activity confers a certain degree of immunization.

